# The Pharmacology and Therapeutic Utility of Sodium Hydroselenide

**DOI:** 10.3390/ijms22063258

**Published:** 2021-03-23

**Authors:** Kavitej Samra, Mathun Kuganesan, William Smith, Anna Kleyman, Robert Tidswell, Nishkantha Arulkumaran, Mervyn Singer, Alex Dyson

**Affiliations:** Bloomsbury Institute of Intensive Care Medicine, Division of Medicine, University College London, Gower Street, London WC1E 6BT, UK; kavitej.samra.15@ucl.ac.uk (K.S.); mathun.kuganesan.17@ucl.ac.uk (M.K.); billy.smith.17@ucl.ac.uk (W.S.); a.kleyman@ucl.ac.uk (A.K.); r.tidswell@ucl.ac.uk (R.T.); n.arulkumaran@ucl.ac.uk (N.A.); m.singer@ucl.ac.uk (M.S.)

**Keywords:** selenium, selenoproteins, sulfide, gasotransmitter, mitochondria, reactive oxygen species

## Abstract

Metabolically active gasotransmitters (nitric oxide, carbon monoxide and hydrogen sulfide) are important signalling molecules that show therapeutic utility in oxidative pathologies. The reduced form of selenium, hydrogen selenide (HSe^−^/H_2_Se), shares some characteristics with these molecules. The simple selenide salt, sodium hydroselenide (NaHSe) showed significant metabolic activity, dose-dependently decreasing ex vivo O_2_ consumption (rat soleus muscle, liver) and transiently inhibiting mitochondrial cytochrome C oxidase (liver, heart). Pharmacological manipulation of selenoprotein expression in HepG2 human hepatocytes revealed that the oxidation status of selenium impacts on protein expression; reduced selenide (NaHSe) increased, whereas (oxidized) sodium selenite decreased the abundance of two ubiquitous selenoproteins. An inhibitor of endogenous sulfide production (DL-propargylglycine; PAG) also reduced selenoprotein expression; this was reversed by exogenous NaHSe, but not sodium hydrosulfide (NaHS). NaHSe also conferred cytoprotection against an oxidative challenge (H_2_O_2_), and this was associated with an increase in mitochondrial membrane potential. Anesthetized Wistar rats receiving intravenous NaHSe exhibited significant bradycardia, metabolic acidosis and hyperlactataemia. In summary, NaHSe modulates metabolism by inhibition of cytochrome C oxidase. Modification of selenoprotein expression revealed the importance of oxidation status of selenium therapies, with implications for current clinical practice. The utility of NaHSe as a research tool and putative therapeutic is discussed.

## 1. Introduction

Basic elemental signaling molecules offer significant potential as novel therapeutics. The most frequently studied is hydrogen sulfide that, alongside carbon monoxide and nitric oxide, constitutes a small group of gaseous molecules, the ‘gasotransmitters’ [1]. The ability of sulfide gas (H_2_S) inhalation to induce a ‘suspended animation-like state’ in mice [2] generated substantial interest in the use of sulfide-liberating agents to modulate oxidative pathologies by reduction of global- or organ-specific metabolism [3]. Initially led by basic sulfur salts and, subsequently, by more complex organic and inorganic molecules, this approach has been a major research focus for more than a decade [4].

Such metabolic modulators can confer protection against reperfusion injury, a consequence of the necessary urgent revascularization of ischemic organs. While underlying pathophysiological mechanisms have yet to be definitively determined, a strong evidence base indicates that rapid restoration of oxygen and substrate to the ischemic tissue drives mitochondrial respiration to generate large quantities of damaging reactive oxygen species (ROS) [5]. This overproduction may be ameliorated with a short-term inhibitor of oxidative phosphorylation while maintaining a level of metabolism that supports cell viability and functionality [6]. This approach extends beyond traditional gasotransmitters, with gases and basic salts of both non-sulfur chalcogens (selenides) [7] and halogens (iodides, bromides) [8] impacting on metabolism and/or exhibiting pre-clinical efficacy in treating oxidative pathologies.

Owing to their proximity in the periodic table, sulfur and selenium share several chemical and biological characteristics. We [9] and others [10] have postulated that the physiological derivative of selenium, hydrogen selenide (as gaseous H_2_Se and anionic HSe^−^), could be an additional member of the gasotransmitter class. Less contentiously, selenium is considered an essential micronutrient [11] that supports diverse physiological roles, most notably as a structural and functional component of antioxidant ‘selenoproteins’ [12]. Thus, selenium and its reduced forms could offer multiple antioxidant mechanisms; as an elemental reducing agent, a modulator of metabolism, and as the catalytic component of specialized enzymes. Here we assessed the impact of sodium hydroselenide (NaHSe) on metabolism and explored a mitochondrial mechanism of action. We also gained novel insights on the pharmacological manipulation of selenoprotein expression, and herein discuss the utility of NaHSe as a research tool and prospective therapeutic.

## 2. Results

### 2.1. Synthesis and Characterization of NaHSe

**Figure 1:**
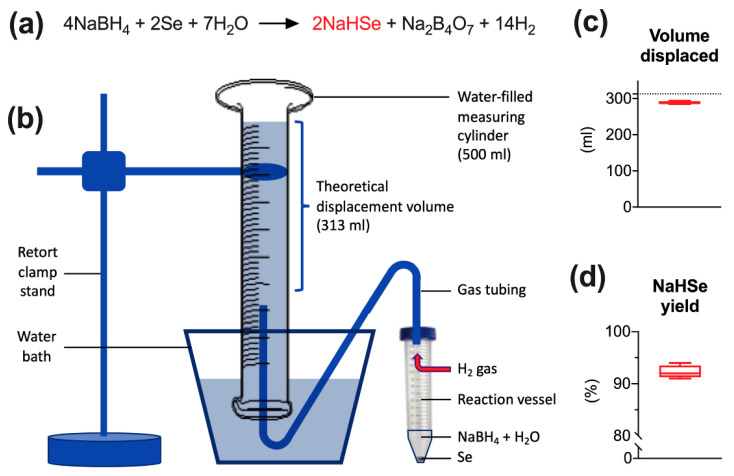
Synthesis of NaHSe. (**a**) Chemical formula. (**b**) Volume displacement apparatus. (**c**) Volume of water displaced by hydrogen gas. (**d**) Estimated yield of NaHSe based on a theoretical maximum water displacement of 313 mL; dotted line in (**c**). Data shown are from four individual experiments (*n* = 4). H_2_, hydrogen gas; H_2_O, water; NaBH_4_, sodium borohydride; Na_2_B_4_O_7_, sodium tetraborate; NaHSe, sodium hydroselenide; Se, selenium.

NaHSe was derived using aqueous sodium borohydride (NaBH_4_) to reduce elemental selenium in a reaction that liberates hydrogen gas (H_2_) (Figure 1a). This gas was diverted to an inverted, water-filled measuring cylinder (Figure 1b), with volume displacement used to estimate the yield of material. For a theoretical displacement volume of 313 mL of water (based on the quantity of precursors and derived using the ideal gas equation), mean (±SEM) volume displacement was 289 ± 2 mL (Figure 1c), corresponding to a NaHSe yield of 92 ± 1% (Figure 1d).

**Figure A1:**
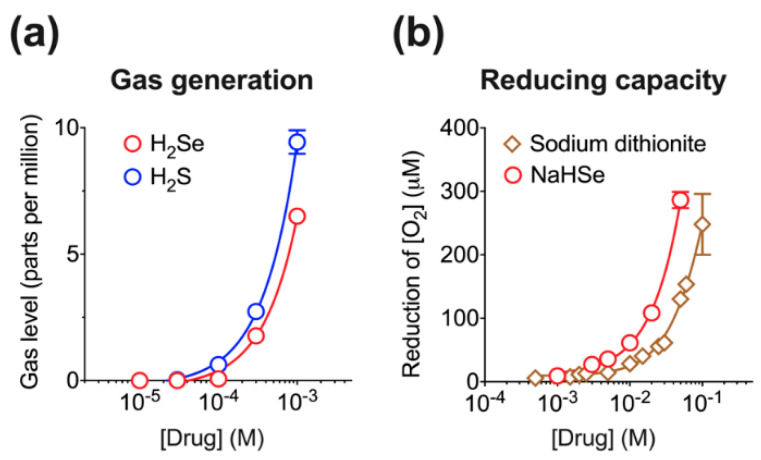
Chemical characteristics of NaHSe. (**a**) Hydrogen selenide gas generation. (**b**) Reducing capacity. Data shown are from three individual experiments (*n* = 3). H_2_S, hydrogen sulfide gas; H_2_Se, hydrogen selenide gas; NaHSe, sodium hydroselenide.

Prior to assessment in biological matrices, basic chemical characteristics of NaHSe were examined, focusing on the generation of gaseous hydrogen selenide (H_2_Se), and the ability of NaHSe to act as a reducing agent. Using an assay optimized for the measurement of hydrogen sulfide [13], we observed concentration-dependent generation of H_2_Se (Figure A1a). A similar pattern was seen with hydrogen sulfide gas (H_2_S; used as a positive control; Figure A1a). The ability of NaHSe to act as a chemical reducing agent was assessed in air-tight chambers using a polarographic oxygen electrode. Concentration-dependent decreases in [O_2_] were observed upon addition of either NaHSe (Figure A1b), or the standard laboratory reducing agent, sodium dithionite (Na_2_S_2_O_4_).

### 2.2. Metabolic Effects of NaHSe Ex Vivo

**Figure 2:**
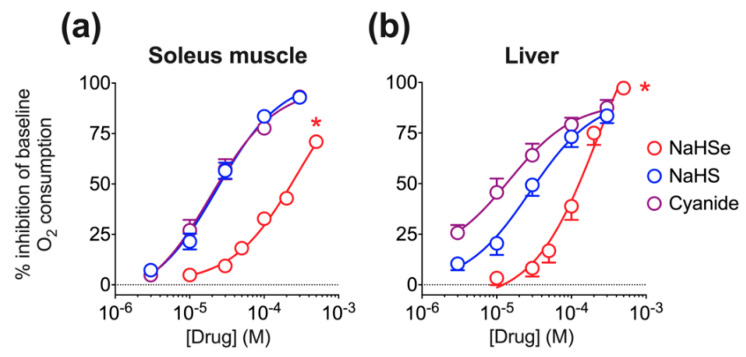
Ex vivo oxygen consumption in (**a**) rat soleus muscle and (**b**) liver tissue homogenate. * *p* < 0.05 vs. NaHS and cyanide, overall ANOVA; *n* = 6–12/group. NaHS, sodium hydrosulfide; NaHSe, sodium hydroselenide.

A polarographic oxygen electrode was used to measure oxygen consumption in dissected rat soleus muscle and homogenized liver tissue. NaHSe inhibited oxygen consumption in a concentration-dependent manner, with IC_50_ (concentration for 50% inhibition) values of 277 µM and 229 µM in soleus muscle (Figure 2a) and liver (Figure 2b), respectively. Sodium hydrosulfide (NaHS) and potassium cyanide, used as positive controls, showed approximately 10–15× greater potency (Figure 2).

**Figure A2:**
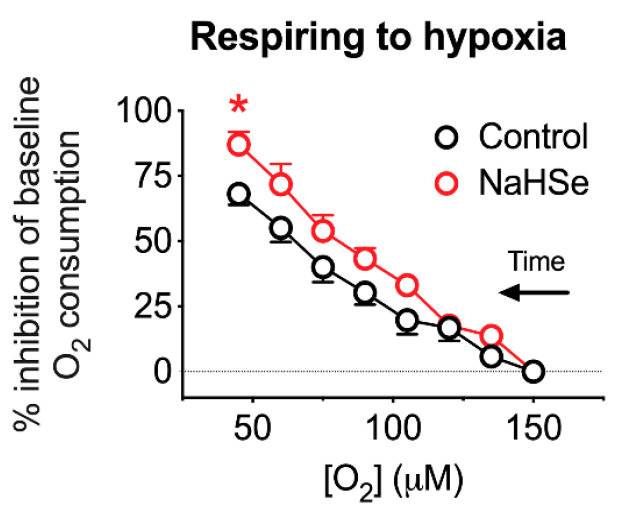
Effect of NaHSe on oxygen consumption by soleus muscle tissue respiring to hypoxia. NaHSe (10 µM) was applied at 150 µM (O_2_). Note that in this model, oxygen consumption decreases below 150 µM (O_2_); this explains inhibition over time in control tissues (standard media only). * *p* < 0.05 (overall ANOVA and at 50 µM [O_2_] with a post-hoc test); *n* = 6/group.

In a separate experiment, the impact of NaHSe on oxygen consumption was assessed in soleus muscle tissue respiring to hypoxia. A concentration of NaHSe (10 µM) that was ineffective in ‘normoxia’ (Figure 2a, where [O_2_] > 150 µM) significantly inhibited oxygen consumption in hypoxic tissues (*p* < 0.05) (Figure A2).

**Figure 3:**
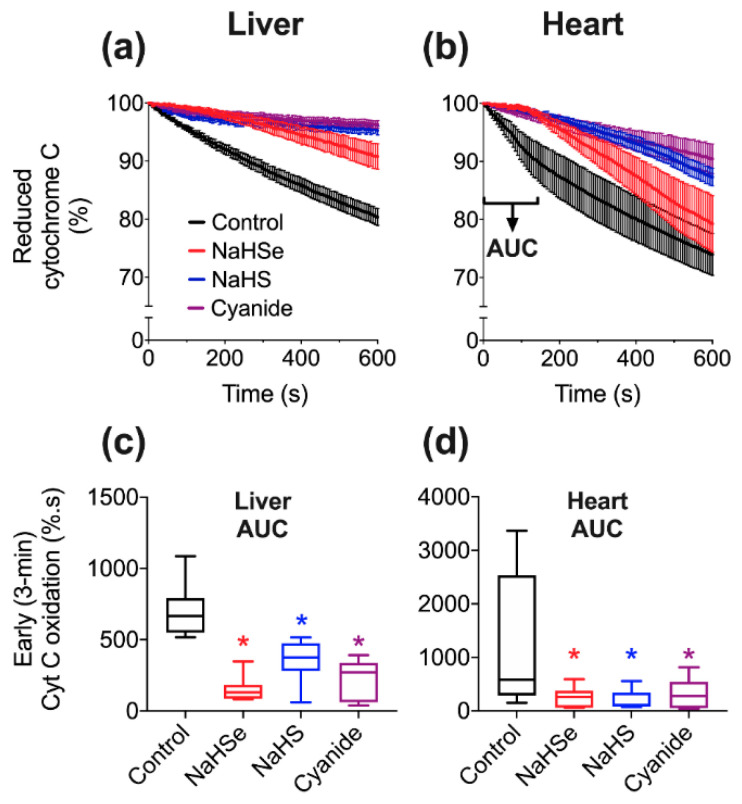
Inhibition of mitochondrial cytochrome C oxidase activity in (**a**) rat liver and (**b**) heart tissue. Panels (**c**,**d**) show area-under-curve (AUC) calculations from the first three minutes of the assay that reflect the oxidation of cytochrome C. * *p* < 0.05 vs. control, one-way ANOVA; *n* = 7–10/group. NaHS, sodium hydrosulfide; NaHSe, sodium hydroselenide.

The mechanism for the observed inhibition of O_2_ consumption by NaHSe was next investigated. As mitochondrial complex IV (cytochrome C oxidase; CCO) is inhibited by NaHS, this mechanism would likely be a strong candidate. Indeed, NaHSe caused transient inhibition of complex IV in both rat liver (Figure 3a) and heart (Figure 3b) homogenate. Area-under-curve (AUC) analysis showed significant inhibition by NaHSe in both tissues in the first three minutes (*p* < 0.05); (Figure 3c,d).

**Figure A3:**
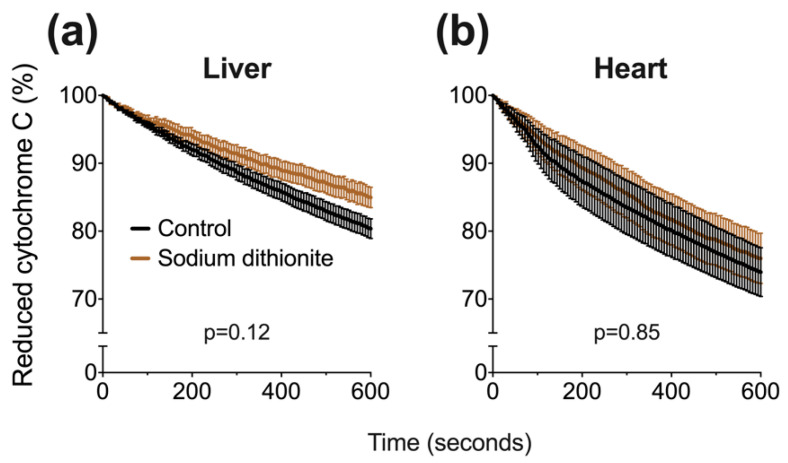
Impact of sodium dithionite on cytochrome C oxidase activity in liver (**a**) and heart (**b**) homogenate. Statistics: 2-way ANOVA; *n* = 7–10/group.

As this mitochondrial complex IV assay is based on measuring the temporal decrement in reduced cytochrome C [14], we were mindful of potential interference from the reducing capacity of NaHSe acting via a chemical rather than a biological mechanism. We thus assessed a concentration of sodium dithionite that could exhibit the same reducing capacity (1 mM) as the NaHSe concentration applied. No significant difference was observed in either liver (*p* = 0.12) or heart (*p* = 0.85) tissue (Figure A3a and Figure A3b, respectively).

### 2.3. In Vitro Modulation of Selenoprotein Expression

**Figure 4:**
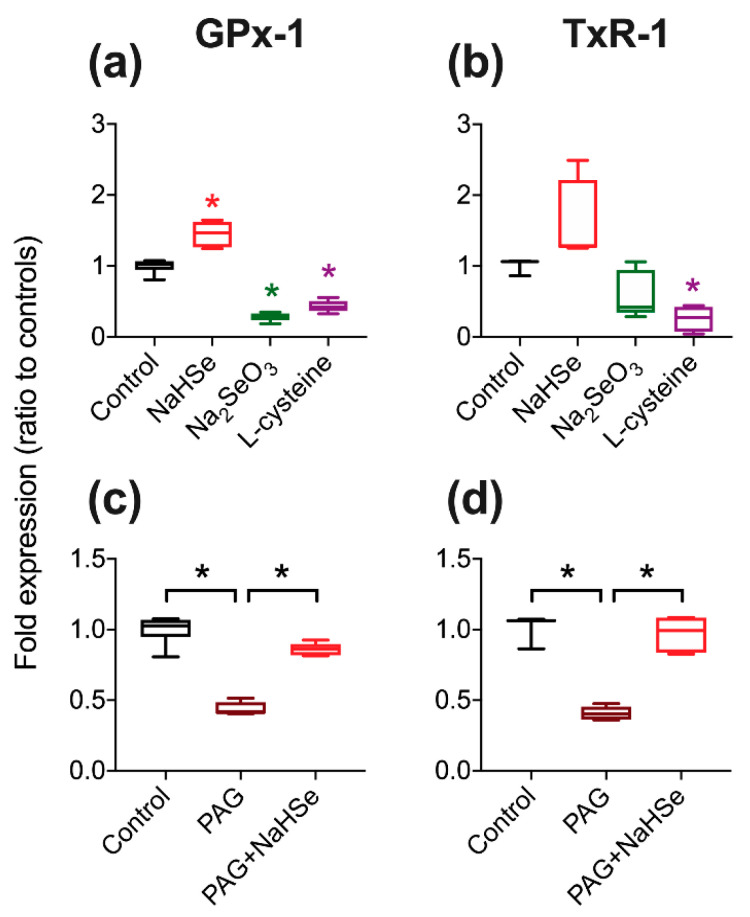
Pharmacological manipulation of selenoprotein expression. Impact of Se- oxidation status on (**a**) GPx-1 and (**b**) TxR-1 protein expression where NaHSe and Na_2_SeO_3_ are the reduced and oxidized forms, respectively. Effects of propargylglycine (PAG) (an inhibitor of endogenous hydrogen sulfide production by [CSE] cystathionine γ-lyase) are shown in panels (**c**,**d**). * *p* < 0.05 vs. control, or between treatments (indicated with brackets), one-way ANOVA; *n* = 3–6/group. NaHSe, sodium hydroselenide; Na_2_SeO_3_, sodium selenite; PAG, dl-propargylglycine.

The currently accepted principal biological action of selenium is its incorporation into selenoproteins. The ability of NaHSe (reduced form) or sodium selenite (Na_2_SeO_3_; oxidized form) to alter the expression of two abundant selenoproteins (glutathione peroxidase-1, GPx-1 and thioredoxin reductase-1, TxR-1) was assessed in HepG2 human hepatocytes, measured by Western blot analysis. NaHSe (1h incubation) significantly (*p* < 0.05) increased GPx-1 expression at 24 h (Figure 4a). Surprisingly, the oxidized form (selenite) showed a reciprocal effect, with a significant (*p* < 0.05) fall in GPx-1 expression relative to non-treated controls. This pattern was mirrored with respect to TxR-1 expression, albeit not significantly (*p* = 0.05 for selenide, *p* = 0.15 for selenite; Figure 4a,b, respectively). The amino acid L-cysteine was used as a positive control to decrease selenoprotein synthesis as it interferes with selenocysteine, the amino acid precursor of hydrogen selenide; a significant reduction was seen in the expression of both selenoproteins (Figure 4a,b).

**Figure A4:**
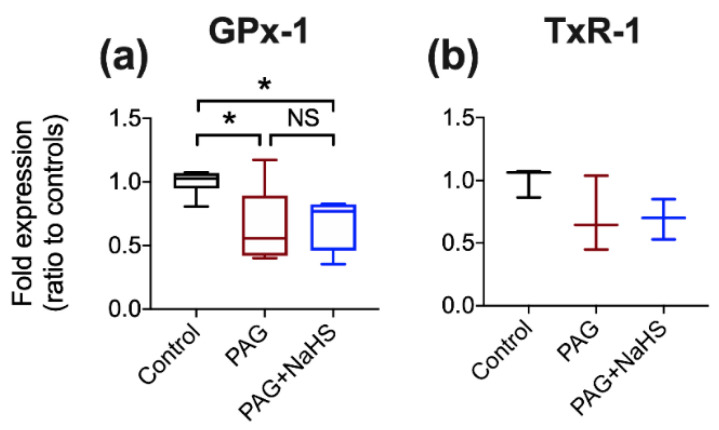
Impact of sulfide mimetics on (**a**) GPx-1 and (**b**) TxR-1 protein expression. * *p* < 0.05 vs. control, or between treatments when indicated with brackets, one-way ANOVA; *n* = 3–6/group. NaHS; sodium hydrosulfide; NS, not significant; PAG, DL-propargylglycine.

DL-propargylglycine (PAG) inhibits cystathionine γ-lyase (CSE), an enzyme responsible for catalyzing the production of endogenous hydrogen sulfide from L-cysteine. The analogous route for endogenous hydrogen selenide production uses selenocysteine, catalyzed by selenocysteine lyase (SCLY). We speculated that PAG could also interfere with the production of endogenous hydrogen selenide by inhibiting SCLY. PAG significantly decreased both GPX-1 and TxR-1 expression (*p* < 0.05; Figure 4c,d), and this could be reversed by the addition of (exogenous) hydrogen selenide (NaHSe). Selenoprotein expression could also be subject to regulation by endogenous hydrogen sulfide; however, addition of (exogenous) sulfide to PAG-treated cells did not impact upon selenoprotein expression (Figure A4).

**Figure A5:**
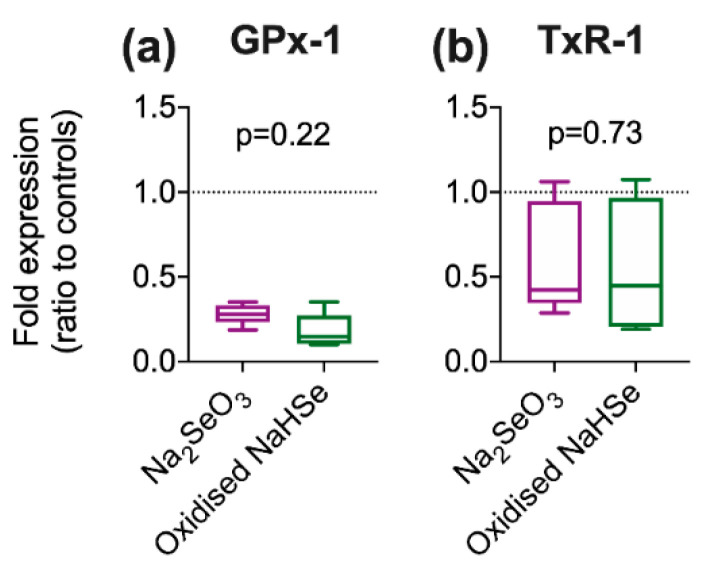
Impact of oxidized seleno-mimetics on (**a**) GPx-1 and (**b**) TxR-1 expression. Statistics: one-way ANOVA; *n* = 5/group. NaHSe, sodium hydroselenide; Na_2_SeO_3_, sodium selenite.

The unexpected decrease in selenoprotein expression with (oxidized) selenite was further examined by comparing commercially purchased material with the oxidation product(s) of NaHSe generated in-house by bubbling with oxygen gas. A comparable reduction in selenoprotein expression was observed in both treatment groups for both selenoproteins (Figure A5).

### 2.4. In Vitro Oxidative Stress Model

**Figure 5:**
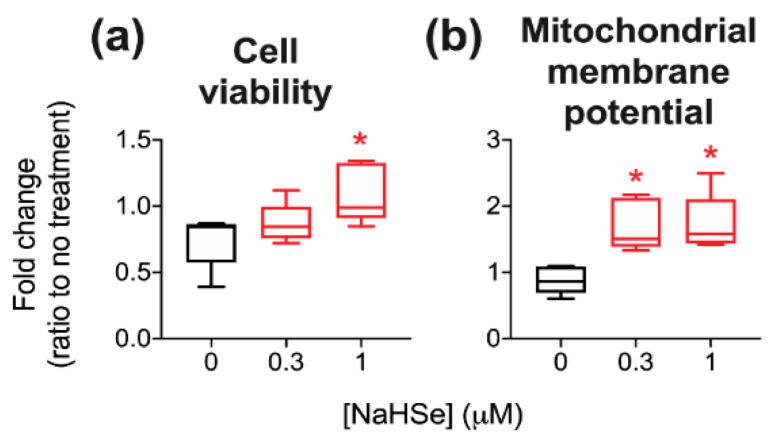
Cytoprotection against hydrogen peroxide with NaHSe, as determined by (**a**) cell viability and (**b**) mitochondrial membrane potential. * *p* < 0.05 vs. control, one-way ANOVA; *n* = 5/group. NaHSe, sodium hydroselenide.

**Figure A6:**
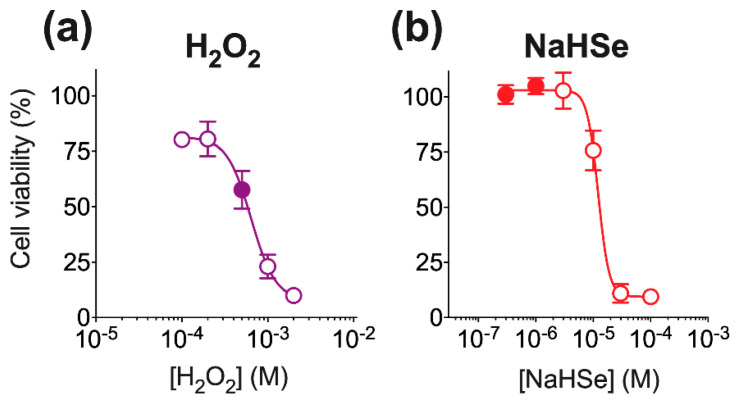
Cell viability following incubation with either (**a**) hydrogen peroxide or (**b**) NaHSe. *n* = 4–7/group. The filled symbols show the concentration of H_2_O_2_ subsequently used for the oxidative stress model (500 µM), and concentrations of NaHSe tested for putative efficacy (0.3 and 1 µM). NaHSe, sodium hydroselenide.

**Figure A7:**
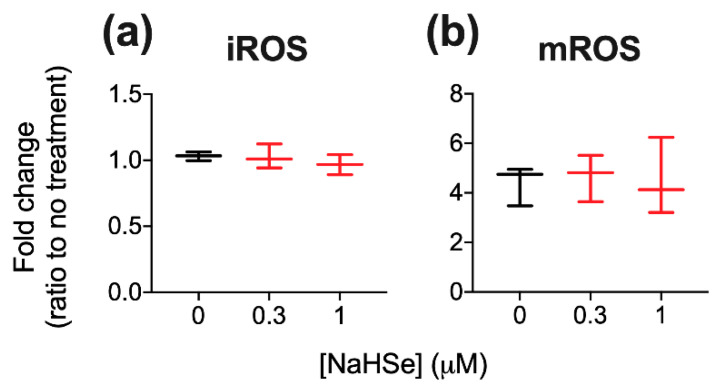
(**a**) Intracellular (i)- and (**b**) mitochondria (m)-derived reactive oxygen species (ROS). *n* = 3/group. NaHSe, sodium hydroselenide.

The therapeutic utility of NaHSe was determined in a HepG2 cell line exposed to hydrogen peroxide (H_2_O_2_). To optimise this assay, the LD_50_ (concentration causing 50% lethality at 24 h post-incubation) of both the insult (H_2_O_2_) and the therapy (NaHSe) were first assessed. LD_50_ values were 639 and 12.2 µM, respectively; concentration–response curves are shown in Figure A6. An oxidative stress model was subsequently established using a concentration of H_2_O_2_ (500 µM) estimated to cause 40% lethality. Hydrogen selenide was used at non-toxic concentrations (0.3 and 1 µM), with allowance for a 10-fold therapeutic window. Incubation with hydrogen selenide for 1h conferred cytoprotection in a concentration-dependent manner (Figure 5a). Mitochondrial membrane potential is an intermediate form of energy storage used by ATP synthase to make ATP. It is also the driving force for transport of ions and proteins necessary for healthy mitochondrial functioning. Mitochondrial membrane potential was increased with both concentrations of NaHSe (Figure 5b). Mitochondrial, but not intracellular, ROS were elevated five-fold over cells receiving no oxidative insult, but levels were unaffected by NaHSe treatment (Figure A7).

### 2.5. In Vivo Pharmacology of NaHSe

**Figure 6:**
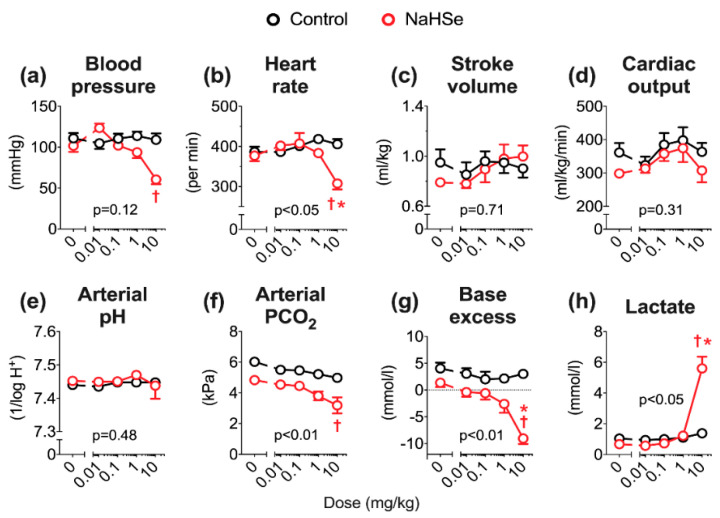
Impact of NaHSe on hemodynamics and myocardial function (panels **a**–**d**), arterial acid-base status and lactate levels (panels **e**–**h**). Statistics: one- or two-way ANOVA; *n* = 5/group. Stated *p*-values are the overall two-way ANOVA; * *p* < 0.05 vs. control, † *p* < 0.05 vs. baseline (shown as ‘zero’). NaHSe, sodium hydroselenide; PCO_2_, partial pressure of carbon dioxide. Base excess is defined as the quantity of acid or base required to titrate a litre of blood back to physiological pH (7.4). Negative values are interpreted as a metabolic acidosis.

Lastly, the in vivo pharmacology of NaHSe was examined using anaesthetized, instrumented and, importantly, normothermic rats. Using a dose-escalation study design (with NaHSe administered as intravenous bolus doses over 1-min), 0.01 mg/kg was the lowest dose of selenide that caused a biologically relevant (~20 mmHg), but not statistically (*p* = 0.07) significant, elevation in blood pressure (Figure 6a). With increasing doses, blood pressure fell; this was statistically significant (*p* < 0.05) at the highest dose level (10 mg/kg). This rise was accompanied by significant bradycardia where heart rate fell by ~25% after administration of the highest dose (Figure 6b). Echocardiographic imaging revealed a 20% rise in stroke volume (albeit without statistical difference from controls) (Figure 6c), thus cardiac output remained unaffected by NaHSe treatment (*p* = 0.31). Arterial pH remained unchanged with NaHSe (Figure 6e) as a respiratory alkalosis (related to hyperventilation and denoted by a lower partial pressure of carbon dioxide; Figure 6f) compensated for a metabolic acidosis (measured as a falling arterial base excess, the metabolic component of acid/base interactions; Figure 6g). Significant hyperlactataemia, reflective in this situation of a greater degree of inhibition of oxidative phosphorylation, was seen at the highest dose of NaHSe (Figure 6h).

## 3. Discussion

Elemental signaling molecules such as hydrogen sulfide, selenides [7], iodides and bromides [8] are being investigated as the bioactive component of novel therapeutics. One indication is reperfusion injury, where a transient reduction of mitochondrial respiration could temper the initial burst in mitochondrial activity (and pathologically excessive ROS production) occurring on abrupt restoration of necessary oxygen and substrate provision during revascularization [15].

Gasotransmitters are endogenously generated and act across diverse physiological systems, including modulation of oxidative metabolism [1]. When delivered exogenously, and usually in excess of their normal physiological range(s), aerobic respiration is decreased by either competitive or non-competitive inhibition of mitochondrial cytochrome C oxidase [16]. The most compelling demonstration was the ability of inhaled H_2_S to induce a ‘suspended animation-like state’ in mice [2]. The same group later showed that inhalation of gaseous selenides and iodides could induce similar effects [7,8].

Mindful of the chemical and biological similarities between sulfur and its chalcogen homologue, selenium [17], we [9] and others [10] postulated that its reduced form, hydrogen selenide, could be an additional member of the gasotransmitter class. To explore this hypothesis, we began experimental work using the basic selenide salt, NaHSe. Our approach has been analogous to early studies on delivery of exogenous sulfide, where the basic salts NaHS and Na_2_S became important research tools and the basis of novel (albeit unsuccessful) therapies [18]. With necessary safety precautions and handling, synthesis of NaHSe is straightforward and inexpensive, and can be used in a range of chemical or biological matrices. It should be noted that reduced selenides are particularly prone to oxidation [17], hence, their handling should always be carried out under anoxic conditions, and all diluents/adjuvants should be purified to remove trace metals that can act as oxidants.

We extended the observation that H_2_Se gas modulates metabolism in mice [7], showing dose-dependent reduction of oxygen consumption with NaHSe in two tissue types (skeletal muscle and liver) taken from rats. The known inhibition of mitochondrial complex IV by similar molecules suggested that this would be a strong candidate as the underlying mechanism and, indeed, this inhibition was demonstrated with NaHSe. Of note, inhibition of oxidative phosphorylation observed with selenide differed from sulfide and cyanide with regard to potency and duration of action. Significantly more selenide was required to inhibit oxygen consumption ex vivo, and its effects on cytochrome c oxidase activity were, perhaps desirably, more transient. With respect to its biological chemistry, selenium is more readily oxidized and more kinetically labile than sulfur [17]; this is likely to explain its transient nature. The greater effectiveness observed during hypoxia, where less selenide is deactivated by oxidation, is supportive of this notion.

In vivo, we previously reported that administration of NaHS is dose-limiting, with a maximum tolerated (intravenous bolus) dose (MTD) of 1 mg/kg [15]. Using the same anesthetized rat model, the MTD of NaHSe was 10 mg/kg; this 10-fold difference is broadly in line with our ex vivo findings. NaHSe could replicate some of the hemodynamic effects of basic sulfur salts, most notably the impact on blood pressure and heart rate. Aside from an initial rise in blood pressure (that could reflect interaction with other vasoactive molecules), NaHSe caused dose-dependent hypotension. Sulfide-induced vasodilation is well recognized, with diverse potential mechanisms including interaction with ion channels, smooth muscle ATP depletion, intracellular acidification, release of lipid mediators and modulation of calcium signaling [19]. While an abrupt decrease in systemic arterial pressure would be expected to induce a baroreceptor-mediated tachycardia [20], our findings here (notably in normothermic rats), and in reports using basic sulfur salts, suggest the opposite [20,21,22]. Sulfide-induced bradycardia occurs despite no changes in QT-interval, vagal tone, or enablement by ion channels [20,22]. The precise molecular mode(s) of action underpinning the hemodynamic effects of selenide, and comparability to sulfide, require further investigation.

Contrary to its hemodynamic effects, the impact of selenide on acid-base balance is more readily explained. No impact was seen on arterial pH, as in these anesthetized but spontaneously breathing animals, a respiratory alkalosis compensated for the metabolic acidosis. We previously observed metabolic acidosis in mechanically ventilated rats receiving a slow-release sulfide donor, and postulated an in vivo mechanism of action at the level of the mitochondrion [15]. When oxidative metabolism is unsustainable, either due to decreased delivery and/or utilization of O_2_, cells increase ATP hydrolysis and their dependency on glycolysis. The observed rise in lactate levels with NaHSe treatment supports a greater reliance on (aerobic) glycolysis following partial inhibition of oxidative phosphorylation, with diversion of pyruvate towards lactate.

Inclusion of any prospective candidate into the gasotransmitter class requires satisfaction of five criteria [1]. Hydrogen selenide can fulfil the first three criteria, being present as a small molecule of gas [17], freely permeable to cell membranes [23], and endogenously generated under regulatory (enzymatic) conditions [9]. The currently accepted function of hydrogen selenide at physiologically relevant concentrations relates entirely to its incorporation into selenoproteins, and there are no recognized cellular effects that operate via second messengers. To date, there are 25 known human selenoproteins, most of which operate as oxidoreductase enzymes [24]. These include families of glutathione peroxidases, thioredoxin reductases and deiodinases. Their dysfunction and/or dysregulation is implicated in diverse pathologies including diabetes, Alzheimer’s disease, cardiovascular diseases and cancer [24,25]. Incorporation of selenium into selenoproteins requires a uniquely adapted translational machinery that utilizes hydrogen selenide derived either from Se-oxidation products, or enzymatically from selenocysteine [9].

Using an in vitro model, the expected increase in selenoprotein expression was noted on exposure to hydrogen selenide. We were however surprised to observe a significant decrease in selenoprotein expression upon incubation with the oxidized Se-product, sodium selenite. The impact of oxidation status of basic elemental salts has been previously noted whereby reperfusion injury (following myocardial ischemia) in mice could be moderated by selenide and iodide (reduced forms), but not by the oxidation product(s) selenite and iodate [7,8]. The oxidation status of Se supplements could have implications for current clinical practice but this has been overlooked [9]. A recent meta-analysis examined 19 clinical trials where intravenous sodium selenite (the oxidized form) was used in acute or critically ill patients [26]; although a modest reduction in overall mortality was recorded, numerous secondary endpoints, including 28-day all-cause mortality and length of stay in intensive care, showed no effect. The oxidized product was selected for use in these studies as it shows lower toxicity, far greater stability and, when used in (healthy) animals, can increase expression of selenoproteins [27]. However, conversion to bioactive hydrogen selenide requires reduction by endogenous antioxidant proteins such as glutathione and thioredoxin. This may not be achievable in patients in whom antioxidant defenses are already strained.

The synthesis and metabolism of (hydrogen) sulfide and selenide show several similarities. Hydrogen sulfide is synthesized by three enzymes; CSE, cystathionine b-synthase (CBS), and 3-mercaptopyruvate sulfur-transferase (3MST), reviewed in detail elsewhere [28]. CSE catalyzes production of hydrogen sulfide from L-cysteine using pyridoxal 5′-phosphate (PLP) as a cofactor. The analogous generation of hydrogen selenide, catalyzed by SCLY from selenocysteine, is also PLP-dependent [29]. PAG, commonly used as a (broadly specific) CSE inhibitor, is reported to act by targeting the PLP binding site of the enzyme [30]. Consequently, we demonstrated that PAG could inhibit selenoprotein synthesis, potentially by PAG/PLP disruption of SCLY. Selenoprotein expression could be rescued by exogenous addition of selenide as this provides substrate for selenoprotein synthesis downstream of SCLY inhibition. However, the concentration of PAG used would also inhibit CSE and decrease endogenous hydrogen sulfide production. This is relevant as (supra)physiological concentrations of sulfide impact on selenoprotein expression [31]. However, using sulfide concentrations that reflect physiological conditions (mid-nanomolar), we found the impact of PAG-induced SCLY inhibition on selenoprotein expression was sulfide-independent. Since PAG also impacts on other enzymes, either PLP- or non-PLP-dependent [30,32], involvement of other pathways cannot be completely excluded. However, given the complete restoration of the abundance of two selenoproteins with selenide in PAG-treated cells, off-target effects appear unlikely. Many studies have used PAG to elucidate the (patho)physiological and/or protective role(s) of endogenous hydrogen sulfide, for example, on cardio-protection [33]. Although the impact of PAG on selenoprotein expression in vivo is yet to be determined, our results suggest the possibility of an unrecognized manipulation of selenoproteins.

A role for NaHSe as a cytoprotective agent was examined using a H_2_O_2_-induced oxidative stress model where a marked improvement in viability was noted in NaHSe-treated cells. Unexpectedly, no difference was seen in intracellular ROS (±NaHSe treatment), since a similar model, albeit in a different cell type, showed a two–three-fold rise [31]. NaHSe could confer cytoprotection by up to three potential mechanisms: as a reducing agent, a metabolic modulator and as the catalytic component of selenoproteins. Our studies do not yet allow robust identification of the precise molecular mode(s) of action. Mitochondrial membrane potential (MMP), with the accumulation of protons in the intermembrane space, drives phosphorylation of ADP by ATP synthase, and is frequently used as a marker of cell functionality. MMP is also subject to regulation by ROS, such that superoxide can activate mitochondrial uncoupling proteins to dissipate the membrane potential and temper ROS production [34]. While we observed a five-fold increase in mitochondrial ROS in H_2_O_2_-treated cells, this was not modified by NaHSe treatment, perhaps due to preservation of MMP by NaHSe. A higher membrane potential for the same quantity of mitochondrial ROS suggests an improvement in ROS handling; however, this requires further study for full clarification.

Our study has several limitations. First, due to the chemical similarities of gaseous sulfide and selenide, we used a H_2_S detector to measure H_2_Se. Notably, the bespoke H_2_Se detector(s) we trialed lacked sensitivity. Consequently, our methodology does not allow for a fully quantitative assessment of H_2_Se, but it could be used in future to assess relative changes with NaHSe as a reference standard. Second, the method used to synthesize NaHSe generates sodium tetraborate as a by-product. However, we are unaware of any studies or biological mechanism(s) where this could impact upon the outcome measures assessed. Third, NaHSe was used as pre-treatment in our cytoprotection study; while this precludes translation to a more clinically relevant scenario (where a treatment is generally applied after the insult), we were mindful that we did not want to quench H_2_O_2_ with the reducing capacity of NaHSe. Future work, in addition to enabling a more complete understanding of the molecular mode of action, should focus on post-treatment (in non-chemical models) and longitudinal testing, preferably with measurement of real-time ROS production. Lastly, we acknowledge that our in vivo assessment of NaHSe is limited; conscious, long-term safety, and efficacy testing of seleno-mimetics are still required, but this should ideally be performed with more promising clinical candidates.

In summary, we provide a significant contribution to selenium pharmacology, demonstrating that the metabolic effects of NaHSe are due to transient inhibition of cytochrome C oxidase. The importance of oxidation status of selenium therapies is further illustrated, with significant implications for clinical practice. As noted, the work performed here is analogous to the first reports examining basic sulfur salts. These were eventually superseded by superior molecules that allowed better control of sulfide delivery [15,35,36], improved targeting to its intended site of action, and at concentrations that better reflect those derived from endogenous sources [4,37]. We postulate the same will hold true for NaHSe; while it will remain an important research tool, its utility as a therapy would likely be hindered by poor pharmacokinetic and safety profiles. While alternative approaches to treat oxidative pathologies remain elusive, intelligent seleno-mimetic drug design could yield more sophisticated pharmacological agents as prospective future medicines.

## 4. Materials and Methods

### 4.1. Synthesis and Characterization of NaHSe

NaHSe was synthesized as previously described [38] on each day of use (Figure 1a). The reaction uses aqueous sodium borohydride (NaBH_4_) to reduce elemental selenium. These precursors and all other reagents were purchased from Sigma-Aldrich (Gillingham, Kent, UK) unless stated otherwise. Briefly, under a fume hood, and in a large Perspex box filled with (inert) argon gas, NaBH_4_ (151.32 mg) was dissolved in (nitrogen-purged) deionized water (2 mL) and added to an open 50 mL Falcon tube containing an excess (>200 mg) of selenium. The reaction visibly generates 313 mL hydrogen gas over approximately 5 s and is mildly exothermic. Once reacted, 1 mL of product was transferred to an Eppendorf tube and centrifuged (14000 rpm for 1 min). This separates unreacted selenium from the supernatant (1M NaHSe) that was transferred to a fresh tube, kept on ice, and diluted (as required) under argon gas using nitrogen-purged (1×) phosphate buffer saline (pH 7.4).

The yield of product was assessed by measuring the quantity of water displaced by hydrogen gas, a by-product generated during NaHSe synthesis. This was performed on four separate occasions as described above. As no other gases (save for small quantities of hydrogen selenide gas) should be evolved during the reaction, this method would give a good approximation of yield. A 500 mL measuring cylinder was filled with water, inverted in a large water-filled container, and held in place with a retort clamp stand. A Falcon tube lid was modified to incorporate air-tight tubing that was fed from the reaction vessel to the measuring cylinder and used to transfer H_2_ gas.

Given the chemical similarities between gaseous sulfide and selenide, we used an assay previously optimized for measurement of hydrogen sulfide [13] to assess hydrogen selenide gas (H_2_Se) generation. This assay relies on the detection of free headspace H_2_Se, measured using a commercially available H_2_S detector (Z900XP, Environmental Sensors, Boca Raton, FL, USA). Briefly, either NaHS (as a positive control) or NaHSe was dissolved in PBS to 10× stock solutions and rapidly diluted (0.5 into 4.5 mL) into airtight 50 mL Falcon tubes containing nitrogen-purged PBS. This was incubated in a water bath for 1 h at 37 °C. Five mL of headspace gas withdrawn over 10 s was passed through the detector. Peak H_2_S values were recorded and used as a non-quantitative surrogate of H_2_Se generation.

The ability of NaHSe to act as a chemical reducing agent was assessed in air-tight chambers using a polarographic Clark-type electrode (Rank Brothers, Bottisham, UK). This was connected to a sealed chamber and maintained at 37 °C with an external, jacketed water heater. Increasing concentrations of either NaHSe (1–50 mM), or the standard laboratory reducing agent, sodium dithionite (Na_2_S_2_O_4_; 0.5–100 mM), were added to the chamber, and the decrease in oxygen concentration was recorded.

Particular care needs to be taken when preparing and using hydrogen selenide. Good safety practices should be maintained, and work should only commence following liaison with divisional safety officers. In particular, fume cupboards or hoods must operate efficiently, and researchers should avoid working alone in the laboratory. These rules are essentially the same as those described for use of hydrogen sulfide [39].

### 4.2. Ethics, Animals and Husbandry

Male Wistar rats (300–400 g body weight) were used to harvest tissues for ex vivo studies, and for in vivo experimentation. All experiments were performed in the UK according to local ethics committee (University College London) and UK Home Office guidelines under the Animals (Scientific Procedures) Act 1986. Animals were purchased from Charles River Laboratories (Margate, UK). All animals were healthy and certified pathogen-free and housed in cages of four individuals on a 12-h light/dark cycle, with food and water ad libitum prior to experimentation. Standard cages and bedding were used with additional materials (tissue paper, cardboard tubes) for comfort and cage enrichment. The ‘actual severity’ classification for all studies is ‘non-recovery’. All animals received peri-operative analgesia (subcutaneous buprenorphine, 0.05 mg/kg; Reckitt Benckiser, Slough, UK). Euthanasia at experiment end was performed either by cessation of the circulation (cardiac biopsy) or intravenous sodium pentobarbitone (Pentoject; Animalcare, York, UK).

### 4.3. Modulation of Metabolism

#### 4.3.1. Oxygen Consumption Experiments

For ex vivo oxygen consumption experiments, soleus muscle or liver samples were obtained from anaesthetized rats. Soleus muscle was dissected and immediately transferred to plastic Petri dishes containing ice-cold biopsy preserving solution (BIOPS; isolation medium). This constituted CaK_2_EGTA (2.77 mM), K_2_EGTA (7.23 mM), Na_2_ATP (5.7 mM), MgCl_2_6H_2_O (6.56 mM), taurine (20 mM), Na_2_ phosphocreatine (15 mM), imidazole (20 mM), dithiothreitol (0.5 mM) and MES monohydrate (50 mM), adjusted to pH 7.4. This method enables storage of tissue with no significant impairment of mitochondrial integrity [40]. The muscle was subsequently dissected with fine forceps and resulting fibres permeabilized with 50 µg saponin in 2 mL isolation medium. Fibres were gently stirred on ice for 20 min. Saponin and metabolites were then removed by washing the fibres three times with ice-cold respiratory medium containing EGTA (0.5 mM), MgCl_2_·6H_2_O (3 mM), K-lactobionate (60 mM), taurine (20 mM), KH_2_PO_4_ (10 mM), HEPES (*N*-(2-hydroxyethyl)piperazine-*N*′-(2-ethanesulfonic acid; 20 mM), sucrose (110 mM) and bovine serum albumin (BSA; 1 mg/mL), adjusted to pH 7.4. Following midline laparotomy, a sample of liver (~1 g) was removed, washed briefly with ice-cold PBS and snap-frozen in liquid nitrogen. The tissue was then pulverized under liquid nitrogen using a pestle and mortar, and kept at −80 °C until use. Sixty mg was then added to 1 mL BIOPS; the homogenate was centrifuged at 7800 rpm for two minutes, and the supernatant was kept on ice until use.

Oxygen consumption was determined using the above Clark electrode. The fall in oxygen concentration within the closed chamber over time was recorded; this was subsequently corrected for drift (oxygen consumption by the electrode), and either the dry weight of each muscle sample or protein content of the liver supernatant. The respiratory medium in the chamber was agitated using a magnetic stirrer. Substrates for mitochondrial respiratory chain complexes I (glutamate, 10 mM; malate, 5 mM) and II (succinate, 5 mM) were added to each chamber by injection through the chamber lid seal. Small bundles (approximately 5 mg) of muscle tissue or 30 µL liver supernatant were then added and the medium oxygenated to approximately 250 μM O_2_ by injecting 3 mL of 100% O_2_ gas.

Baseline oxygen consumption within the chamber was assessed over two minutes followed by addition of increasing concentrations of NaHSe (10–500 μM), NaHS or K-cyanide (both 3–300 μM). Concentrations were increased at 2-min intervals, with oxygen consumption measured in the last minute of each interval. All ‘normoxia’ experiments were performed between 250 and 150 μM O_2_, below which the level of oxygen consumption becomes dependent on the O_2_ concentration within the chamber.

In a second experiment, a single concentration of NaHSe (10 μM) was used. This concentration was ineffective during normoxia, with soleus muscle tissue allowed to respire to hypoxia. Respiratory medium was used as a control. Oxygen consumption was determined at 150 μM (baseline) and after the addition of NaHSe, at 15 μM [O_2_] gradations until the concentration fell to 45 μM O_2_.

#### 4.3.2. Cytochrome C Oxidase Activity

A well-established assay [14] was used (with modifications) to assess CCO activity in liver and heart tissue. Briefly, 12.5 mg cytochrome C was added to 1 mL of 20 mM potassium phosphate buffer, followed by 1 mg of sodium dithionite to fully reduce cytochrome c. The reduction of cytochrome C was assessed by absorbance spectrophotometry using a microplate reader and BioTek (Gen5) software (Synergy 2, North Star Scientific, Sandy, UK). The ratio of absorbance at 550 and 565 nm was confirmed to be above 6, signifying an acceptable proportion of reduced cytochrome C. Next, a master-mix was assembled, comprising 5 parts potassium phosphate buffer (100 mM), 3.6 parts distilled water and either 0.4 parts tissue or BIOPS solution. 80 μL of either tissue-positive or negative mix was added to a 96-well plate followed by 10 μL of drugs. We used the IC_50_ value from our oxygen consumption experiments in liver tissue (Figure 2) for either NaHSe, NaHS or K-cyanide. BIOPS (10 μL) served as a negative control. We additionally tested sodium dithionite (at twice the NaHSe concentration; 458 μM) to ensure CCO activity was not artificially stalled by the reducing capacity of these drugs. Enzyme activity was initiated with the addition of 10 μL reduced cytochrome C, with (decreasing) absorbance (550 nm) measured every 6 s for 10 min.

### 4.4. In Vitro Studies

Human hepatoma (HepG2) cells were cultured to 80% confluence in Dulbecco’s modified Eagle medium (DMEM), supplemented with 10% fetal bovine serum and 1% penicillin/streptomycin (37 °C, 5% CO_2_). Cells were seeded on 24-well plates in triplicate at a density of 1.5 × 10^6^ cells/mL. Cells were incubated for a further 24h before being assigned to separate studies.

#### 4.4.1. Pharmacological Manipulation of Selenoprotein Expression

Cells were treated with either standard medium (control), NaHSe (30 nM), sodium selenite (Na_2_SeO_3_; 30 nM), L-cysteine (0.1 mM) or DL-propargylglycine (PAG; 2 mM) for 1h. In a subset, cells were pre-treated (for 30 min) with PAG before addition of either NaHSe or NaHS (30 nM). Cells were then washed, the medium replaced, and allowed to incubate under standard conditions for a further 24 h. Protein was extracted in radio-immunoprecipitation assay (RIPA) buffer and concentrations determined using bicinchoninic acid assay (Pierce™ BCA Protein Assay Kit). Western blot was performed on 12% SDS-PAGE gel with 40 μg protein per well. Proteins were transferred to a polyvinylidene difluoride (PVDF) membrane (GE Healthcare, Amersham, UK) at 10V for 45 min, blocked with 5% BSA, and incubated overnight at 4 °C with primary antibodies (GPx-1; 1:1000, Bio-Techne, Abingdon, UK) or thioredoxin reductase-1 (TxR-1; 1:1000, Cell Signalling, London, UK) and alpha-tubulin (1:3000; Cell SignalFling Technology, Danvers, MA, USA). Secondary antibodies were incubated for 1 h at room temperature (1:3000; Agilent Technologies, Santa Clara, CA, USA). The blots were then detected using an enhanced chemiluminescence detection system (GE Healthcare, Chicago, IL, USA) and ratio-metric expression determined using Image Studio Lite (LI-COR, Cambridge, UK).

#### 4.4.2. Viability

Cell viability was determined in tetrazolium chloride (TTC; 1% *w*/*v*)-stained cells [41]. Cells were treated for 24 h with cell medium (DMEM; negative control), or different concentrations of either NaHSe (0.3–100 μM) or hydrogen peroxide (50–2000 μM). These pilot studies aimed to establish a non-toxic concentration range for NaHSe (for use in all of our in vitro studies), and for optimization of an oxidative insult with H_2_O_2_. A positive control was provided by heat-killing the cells at 65 °C for 30-min. Cells were incubated for 1h with colourless TTC, which is enzymatically converted to a red formazan product by living cells. Cells were then lysed using ice-cold (1×) RIPA buffer agitated on an orbital shaker for 1.5 h, then centrifuged (12000 rpm for 10-min). The supernatant (300 μL) was transferred to a 96-well plate and absorbance measured at 494 nm using the above microplate reader. The results were normalized for protein content (using a BCA assay kit; Abcam, Cambridge, UK) and calibrated using internal positive and negative controls.

#### 4.4.3. Oxidative Stress Model

Oxidative stress was induced by 500 μM H_2_O_2_, a concentration that produced ~40% cell death in pilot studies (Figure A6). Cells receiving NaHSe were pre-treated with either 0.3 or 1 μM for 60 min. The cells were then washed, H_2_O_2_ added and incubated for 24 h under standard cell culture conditions. Viability was then measured as above. A subset of cells was allocated to flow cytometry experiments.

#### 4.4.4. Flow Cytometry

Cells were incubated with either tetra-methyl-rhodamine methyl ester (TMRM; 25 nM; Thermo Fischer Scientific, Waltham, MA, USA), 2′,7′-dichlorofluorescein diacetate (H_2_DCFDA; 5 μM; Thermo Fischer Scientific, Waltham, MA, USA) or MitoSox^TM^ (2.5 μM; ThermoFisher Scientific, Waltham, MA, USA) to determine mitochondrial membrane potential [42], intracellular ROS [43] or mitochondrial [44] ROS, respectively. The contents of each well were then transferred to FACS (fluorescence-activated cell sorting) tubes and fluorescence measured using a flow cytometer (LSR Fortessa, BDBiosciences, San Jose, CA, USA). Data were collected and analysed using FlowJo v10.6.1 software (FloJo, Ashland, OR, USA).

### 4.5. In Vivo Studies

Ten animals were anaesthetized as above. They were placed on a heated mat (Harvard Apparatus, Cambridge, UK) to maintain rectal temperature at 37 °C. The left common carotid artery and right internal jugular vein were cannulated using 0.96 mm outside diameter PVC tubing catheter (Scientific Commodities Inc., Lake Havasu City, AZ, USA). The arterial line was connected to a pressure transducer (Powerlab; AD Instruments, Chalgrove, UK) for continuous monitoring of mean arterial blood pressure and intermittent blood sampling, and the venous line was used for administration of fluids and drugs.

Following surgery and a 30-min stabilization period, animals received increasing intravenous bolus doses of NaHSe (0.01–10 mg/kg) or an equivalent quantity (1 mL/kg) of PBS. The diluent was purged with nitrogen prior to use and each dose administered (within 2-min of preparation). Measurements were collected at baseline, then as follows after each dose: blood pressure changes at 30-s to 1-min, cardiac function (by echocardiography; see below for details) within 2 min, and subsequent arterial blood gas analyses (to measure partial pressure of CO_2_ (PCO_2_), lactate, and acid/base changes) at 13 min. Doses were escalated every 15 min.

Transthoracic echocardiography was performed using a 14 MHz probe scanning at 0–2 cm depth (Vivid 7 Dimension, GE Healthcare, Bedford, UK). Aortic blood flow velocities were determined in the aortic arch using pulsed-wave Doppler. Stroke volume was determined as the product of velocity-time integral (VTI) and vessel cross-sectional area. Heart rate was determined by measuring the time between cardiac cycles. Cardiac output was calculated as the product of stroke volume and heart rate.

### 4.6. Data and Statistics

Data are presented as mean ± standard error or median, quartiles and range. Parametric data were analyzed using either repeated measures one- or two-way ANOVA followed by Sidak’s post-hoc test, as appropriate. Non-parametric data were analyzed using the Mann-Whitney U-test. Linear regression was performed using three- or four-parameter inhibitor/response models with least squares fitting method. All statistical analyses were two-tailed and performed using Prism 8.4.3 software (GraphPad Software, San Diego, CA, USA). Multiplicity-adjusted *p*-values < 0.05 were considered statistically significant.

## Data Availability

Upon reasonable request to the corresponding author.

## Abbreviations

ANOVA	Analysis of variance
AUC	Area under curve
BCA	bicinchoninic acid assay
BIOPS	Biopsy preservation solution
BSA	Bovine serum albumin
CaK_2_EGTA	Calcium potassium ethylene glycol-bis(2-aminoethylether)-*N N N*′*N*′-tetra-acetic acid
CCO	cytochrome C oxidase
CSE	Cystathionine γ-lyase
DMEM	Dulbecco’s modified Eagle medium
GPx-1	Glutathione peroxidase-1
FACS	Fluorescence-activated cell sorting
HS^−^	Hydrosulfide ion
HSe^−^	Hydroselenide ion
H_2_	Hydrogen gas
H_2_DCFDA	2′,7′-dichlorofluorescein diacetate
H_2_O	Water
H_2_O_2_	Hydrogen peroxide
H_2_S	Hydrogen sulfide gas
H_2_Se	Hydrogen selenide gas
HEPES	*N*-(2-hydroxyethyl)piperazine-*N*′-(2-ethanesulfonic acid)
IC_50_	Concentration causing 50% inhibition
iROS	Intracellular reactive oxygen species
K_2_EGTA	Potassium ethylene glycol-bis(2-aminoethylether)-*N N N*′*N*′-tetra-acetic acid
K-cyanide	Potassium cyanide
KH_2_PO_4_	Potassium dihydrogen phosphate
K-lacto-bionate	Potassium lacto-bionate
LD_50_	Concentration (dose) causing 50% lethality
MES monohydrate	4-Morpholineethanesulfonic acid monohydrate
MgCl_2_·6H_2_O	Magnesium chloride hexahydrate
MMP	mitochondrial membrane potential
mROS	Mitochondrial reactive oxygen species
Na_2_ATP	Adenosine 5′-triphosphate disodium salt
Na_2_ phosphocreatine	Phosphocreatine disodium salt
NaBH_4_	Sodium borohydride
NaHS	Sodium hydrosulfide
NaHSe	Sodium hydroselenide
Na_2_B_4_O_7_	Sodium tetraborate
Na_2_S	Sodium sulfide
Na_2_S_2_O_4_	Sodium dithionite
Na_2_Se	Sodium selenide
Na_2_SeO_3_	Sodium selenite
PAG	dl-propargylglycine
PCO_2_	Partial pressure of carbon dioxide
PVC	Polyvinyl chloride
PVDF	Polyvinylidene difluoride
RIPA	Radioimmunoprecipitation assay (buffer)
ROS	Reactive oxygen species
RPM	Revolutions per minute
SCLY	Selenocysteine lyase
Se	Selenium
SEM	Standard error of the mean
TMRM	Tetra-methyl-rhodamine methyl ester
TTC	Tetrazolium chloride
TxR-1	Thioredoxin reductase-1
RPM	Revolutions per minute
SCLY	Selenocysteine lyase
Se	Selenium
SEM	Standard error of the mean
TMRM	Tetra-methyl-rhodamine methyl ester
TTC	Tetrazolium chloride
TxR-1	Thioredoxin reductase-1
